# Analysis of Medical Response Team Interventions and the Impact of Certified Training on the Treatment of Patients with Hypoglycaemia—A Simulation Study

**DOI:** 10.3390/jcm14238318

**Published:** 2025-11-23

**Authors:** Damian Krysiak, Michał Ćwiertnia, Michał Wójcik, Piotr Babik, Łukasz Suchanek, Filip Jaskiewicz, Joanna Trojak-Piętka, Michał Szlagor, Wioletta Pollok-Waksmańska, Marek Kawecki, Tomasz Ilczak

**Affiliations:** 1 Department of Emergency Medicine, Faculty of Health Sciences, University of Bielsko-Biala, Willowa 2, 43-309 Bielsko-Biała, Poland; dkrysiak@ubb.edu.pl (D.K.); miwojcik@ubb.edu.pl (M.W.); pbabik@ubb.edu.pl (P.B.); lsuchanek@ubb.edu.pl (Ł.S.); jpietka@ubb.edu.pl (J.T.-P.); mszlagor@ubb.edu.pl (M.S.); mkawecki@ubb.edu.pl (M.K.); tilczak@ubb.edu.pl (T.I.); 2 European Pre-Hospital Research Network, Nottingham NG11 8NS, UK; 3 Department of Emergency Medical Services, Medical University of Lodz, 90-419 Lodz, Poland; filip.jaskiewicz@umed.lodz.pl; 4 Department of Public Health, Faculty of Health Sciences, University of Bielsko-Biala, Willowa 2, 43-309 Bielsko-Biała, Poland; wwaksmanska@ubb.edu.pl

**Keywords:** hypoglycaemia, emergency medicine, paramedics, medical response team

## Abstract

**Background/objectives:** The effectiveness of emergency medical procedures administered to a patient in a life-threatening condition depends, to a large degree, on the knowledge and skills of medical response team personnel. Their competencies can be developed through participation in training and then verified during emergency medicine championships. **Methods:** The research was conducted on the basis of one of the tasks carried out during the ‘16th International Winter Championships in Emergency Medicine’. The task was completed by 28 Polish emergency response teams from ambulance stations across the country. The teams carried out a simulated scenario related to procedures with a patient with hypoglycaemia. The teams’ interventions were assessed in accordance with European Resuscitation Council (ERC) guidelines by judges selected from among academic lecturers and ERC instructors. **Results:** The research showed that 86% of the teams obtained the maximum number of points for adhering to safety procedures. Further, 61% of the teams obtained the maximum of 6 points for the initial assessment, with the average number of points obtained by the teams being 5.54. The average number of points for the physical examination was 21.04, with only one team obtaining the maximum result of 26 points. Additionally, 57% of the teams obtained the maximum number of 6 points for the medical consultation, with the average obtained by the teams being 5.43. The teams obtained, on average, 8.18 points for the correct treatment of hypoglycaemia, with 68% of the teams obtaining the maximum of 9 points. The research demonstrated a positive correlation between the quality of patient examination and the collection of medical data, and the effectiveness of hypoglycaemia treatment. It was also shown that if the team leader had completed an ALS course, they obtained higher scores for the treatment of hypoglycaemia, although this finding is specific to this scenario. **Conclusions:** The teams demonstrated generally high performance in a simulated hypoglycaemia scenario. More complete assessment and history-taking were associated with higher treatment scores. Correct treatment was achieved in 79% of ALS-led teams versus 44% of non-ALS teams, although this observation is specific to this simulation and should not be generalised.

## 1. Introduction

Modern emergency medicine, especially pre-hospital care, requires medical personnel to continually improve their qualifications. In dynamically changing clinical conditions, systematic training and participation in certified courses are key elements in ensuring the high quality of medical interventions. Prior research has shown that paramedics who have participated in advanced courses, such as Advanced Life Support (ALS), Advanced Cardiovascular Life Support (ACLS), Prehospital Trauma Life Support (PHTLS), International Trauma Life Support (ITLS) and Tactical Combat Casualty Care (TCCC), obtain better results in recognising emergencies, taking clinical decisions and effectively implementing procedures [1,2,3]. Such training, conducted in conditions close to real-life conditions, develops skills that can have an important impact on improving patient safety. Research has also shown that regular participation in training courses provides better self-confidence and better coordination of teamwork, especially in stressful situations [1,3]. The impact of medical personnel’s participation in certified training on the quality of emergency care interventions has been evaluated in only a few studies in Poland. The obligation for continuing education among paramedics is implemented in Poland in five-year educational cycles. Within this period, each paramedic is obliged to participate in a 48 h training course, as well as take part in conferences, training, and competitions, thus obtaining the appropriate number of educational points [4]. Emergency medicine championships also allow an individual’s state of knowledge and skills to be verified and provide the opportunity to assess the impact of self-education on the quality and effectiveness of emergency interventions. These events, which attract teams from around the country and across the world, are a key source of data for research into raising the standards of emergency medical care, including the treatment of hypoglycaemia [5].

This research presents an analysis of the performance of emergency medical response teams in a simulated scenario of a patient with hypoglycaemia during the ‘International Winter Championships in Emergency Medicine’. Although hypoglycaemia is not one of the most complex clinical conditions, its rapid diagnosis and suitable treatment can be of crucial importance for a patient’s prognosis. What is more, this situation is good for assessing paramedics’ general competencies, such as correct assessment of a patient’s condition, making decisions regarding treatment, and whether they follow procedures in accordance with current guidelines. Evaluation was also made of the impact of certified training in advanced life support (ALS) on the quality and effectiveness of emergency medical procedures.

Simulation-based assessment is commonly interpreted through established educational frameworks such as Miller’s pyramid of clinical competence and the Kirkpatrick model of training evaluation. While written tests or self-reported confidence primarily reflect cognitive or attitudinal levels (Miller’s “knows”/“knows how”), high-fidelity team simulations allow direct observation of the “shows how” level—behavioural performance in dynamic clinical conditions. From an evaluative perspective, simulation performance also aligns with Level 2 (learning) and partially Level 3 (behaviour change) of the Kirkpatrick model. Embedding this study within these frameworks provides a theoretical lens for understanding how structured assessment and prior training may influence observable team behaviours.

Therefore, the primary objective of this study was to evaluate the quality and variability of clinical performance among emergency medical teams during a standardised hypoglycaemia simulation scenario, with particular attention to the relationship between elements of structured assessment (ABCDE approach and the SAMPLE interview) and treatment accuracy. A secondary aim was to explore whether prior completion of formal, certified training courses (such as ALS) was associated with specific domains of performance. Rather than testing a strict causal hypothesis, this study sought to generate evidence about performance patterns and identify pedagogically relevant gaps that may inform curriculum development.

## 2. Materials and Methods

### 2.1. Design

The research was conducted on the basis of written permission of the Director of the Emergency Medical Service in Bielsko-Biala during the ‘16th International Winter Championships in Emergency Medicine’, which took place on 25–27 January 2022. Permission for the research was obtained from the Ethics Committee of the University of Bielsko-Biała, no. 2025/04/10/E/13. Medical response teams from Poland took part in the championships. Due to the COVID-19 pandemic, teams from abroad did not participate. According to the competition regulations, medical response teams acted on the basis of European Resuscitation Council guidelines and had identical equipment as that found in Polish ambulances, excluding devices for mechanical chest compressions. The performance of each team was evaluated using a structured clinical performance scoring tool, described in detail in Section 2.4.

After the simulated task, each participant completed an anonymous questionnaire containing closed questions (single and multiple responses) and open questions. The questionnaire contained 9 questions related to: gender, age, place of residence, education, profession, work experience and place of work, and completion of a course in advanced life-saving (ALS) on adults certified by the ERC. The time required for completion of the questionnaire was not assessed. All participants agreed to participate in the research. Once completed, the questionnaires were collected by the researcher.

### 2.2. Participants

In total, 84 people participating in the ‘16th International Winter Championships in Emergency Medicine’ qualified to take part in the research (28 three-person medical response teams). Due to the restrictions related to the COVID-19 pandemic, teams from abroad did not participate, despite the fact that the competition is an international event.

The teams comprised representatives of three professional categories: paramedics, system nurses and people with both of these professional titles. The inclusion criteria were qualifications authorising the conducting of emergency medical care, irrespective of the education pathway (further education, bachelor’s or master’s), as well as the requirement of being currently professionally active. This was defined as employment in the State Medical Response Team (MRT) system, Hospital Emergency Departments (ER), admission rooms or other hospital wards in which emergency medical care is provided. The assessment card was completed by evaluating the work of the whole team.

A team representing the organiser took part in the competition, but was not taken into consideration in the general classification and was excluded from participation in the research.

### 2.3. Data Collection

The assessment cards were completed on an ongoing basis by the team of judges during the performance of the simulated task and then checked by the research organiser. The questionnaires were completed by individual team members after finishing the task.

The data was carefully prepared and entered into an Excel spreadsheet, which was password-protected to ensure the protection of the participants’ privacy. Additionally, all the data was anonymised, making it impossible to identify the research participants. After the data had been entered into the matrix, it was passed on for further statistical analysis, which enabled objective conclusions to be drawn on the basis of the research results.

### 2.4. Clinical Performance Scoring Tool

The clinical performance of each Medical Response Team (MRT) was evaluated using a structured scoring tool specifically adapted for the hypoglycaemia scenario. The checklist was developed on the basis of the 2021 European Resuscitation Council (ERC) Guidelines and the standardised ABCDE and SAMPLE algorithms that form the core of pre-hospital patient assessment. To ensure comprehensive evaluation, the tool comprised five domains that reflect the essential components of clinical decision-making in emergency care: (1) assessment of the scene, (2) initial assessment, (3) structured physical examination according to the ABCDE approach, (4) medical history taking using the SAMPLE framework, and (5) diagnosis and treatment.

Each domain contained several observable clinical actions operationalised as checklist items. The weighting of items ranged from 1 to 6 points, depending on their complexity and clinical significance. The maximum achievable score was 50 points. This weighting structure was aligned with the relative importance of specific actions in the management of hypoglycaemia (e.g., accurate glycaemic assessment, airway management, and appropriate glucose administration).

The scoring tool was initially constructed by two experienced paramedics and ERC ALS instructors from the organising committee, both of whom also served as academic teachers. It was subsequently reviewed, modified and approved by a multidisciplinary panel consisting of seven judges: paramedics with many years of field experience, ERC ALS instructors and academic teachers involved in emergency medical education. The tool had been used in previous editions of the emergency medicine championships and was adapted for the present hypoglycaemia scenario.

Before the competition, all judges participated in a structured calibration session during which each checklist item, scoring rule and expected performance standard was discussed. During the event, each team was evaluated live and independently by all seven judges. After each scenario, discrepancies between individual assessments were resolved by consensus. This process—expert-based development, shared pre-assessment calibration, independent multi-rater scoring, and consensus resolution—is a recognised methodological standard for ensuring both content validity and rating consistency in simulation-based Emergency Medical Services (EMS) research.

Although no formal psychometric testing (e.g., inter-rater correlation coefficients) was performed, the combination of expert review, grounding in established clinical guidelines, prior operational use, and multi-rater consensus provides a robust level of methodological adequacy appropriate for exploratory simulation research in pre-hospital emergency medicine.

### 2.5. Statistical Methods

An MS Excel spreadsheet was used for statistical processing of the results, as well as the statistical software Statistica v.13 PL from TIBCO Software Inc., Palo Alto, USA. The quantitative variables were presented using the arithmetic mean, minimum and maximum values, and the standard deviation. The qualitative variables were presented using numerical values and percentages. Comparisons of the dependencies between selected parameters were conducted using the χ^2^ independence test. Comparisons of the dependencies between selected parameters were conducted using the U Mann–Whitney test. The Pearson linear correlation coefficient was used to measure the correlation between the analysed quantitative variables. Values of *p* < 0.05 were accepted as statistically significant.

## 3. Results

Table 1 presents the characteristics of the study group.

Table 2 presents descriptive statistics related to the results from the teams’ assessment cards.

**Figure 1:**
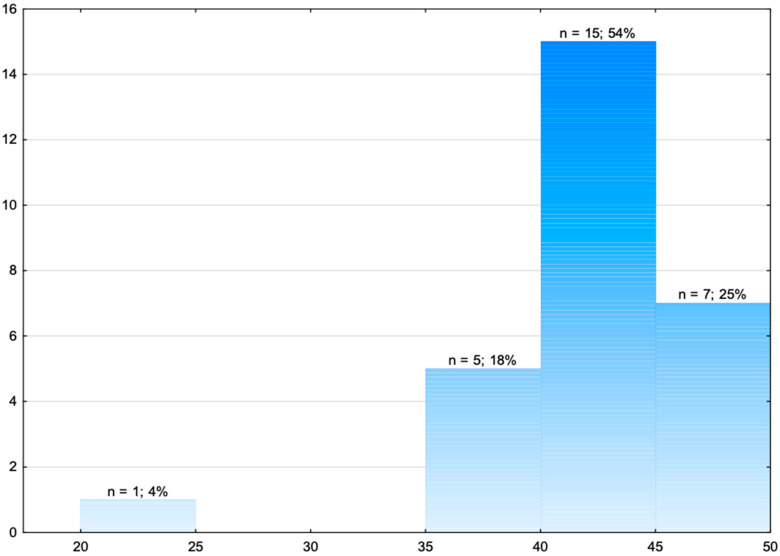
Distribution of teams according to points obtained in individual areas.

Figure 1 presents the distribution of the teams according to the points obtained in individual areas.

The research showed that none of the medical response teams obtained the maximum number of 50 points. The average number of points was 42.82 (±4.96). One team obtained the lowest number of points (23), and one MRT obtained the highest number of points (49).

**Figure 2:**
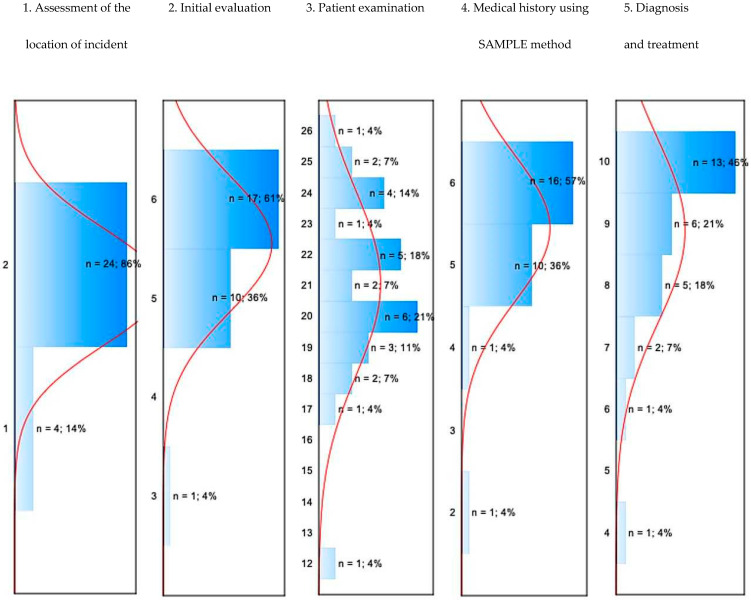
Distribution of the teams according to the points obtained in individual task areas.

Figure 2 presents the distribution of the teams according to the points obtained in individual task areas.

The research showed that the average number of points obtained for assessing the location of the incident was 1.86 (±0.36). The majority of the MRTs (86%) obtained the maximum number of points. The average points for the initial evaluation were 5.54 (±0.69). 61% of the teams obtained the maximum number of points (Figure 2). 36% of teams obtained 5 points, and 1 team obtained 3 points. For the examination of the patient, the average number of points was 21.04 (±2.95). One team obtained the maximum number of 26 points. Most teams (21%) obtained 20 points. The average number of points obtained for collecting the medical history according to the SAMPLE method was 5.43 (±0.88), and 57% of teams obtained the maximum of 6 points. For diagnosis and treatment, the teams obtained on average 8.96 points (±1.53). 46% of the teams obtained the maximum of 10 points in this regard, with the remaining teams obtaining between 4 and 9 points (Figure 2).

**Figure 3:**
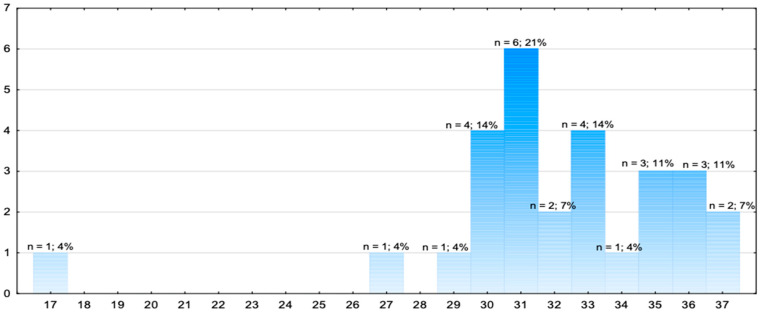
Distribution of the points obtained by the teams in terms of the ABCDE examination and the collection of medical history.

Table 3 and Figure 3 present an assessment of the teams in terms of examination of the patient using the ABCDE method and the collection of the medical history.

The research showed that none of the teams obtained the maximum number of 38 points for the ABCDE examination and the collection of medical history. The highest number of points (37) was obtained by 2 teams. 21% of the response teams obtained 31 points. The lowest number of points obtained by a team was 17. 

**Figure 4:**
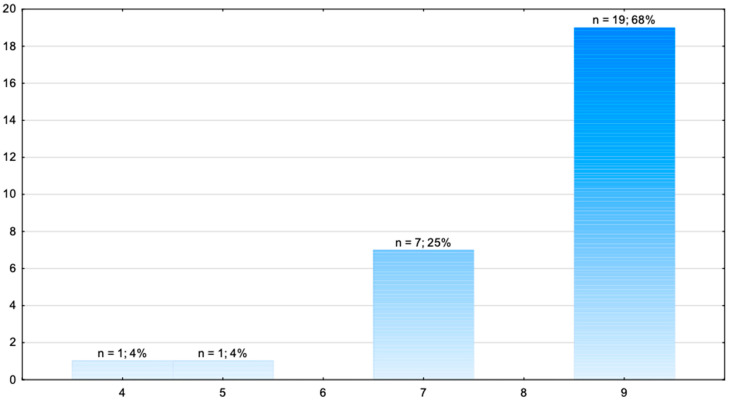
Distribution of the points obtained by the teams in terms of the correct treatment of hypoglycaemia.

Table 4 and Figure 4 present an assessment of the teams with regard to the correct treatment of hypoglycaemia.

The research showed that 68% of the medical response teams obtained the maximum number of points for the treatment of hypoglycaemia. One team obtained 4 points. The average number of points was 8.18 (±1.36).

**Figure 5:**
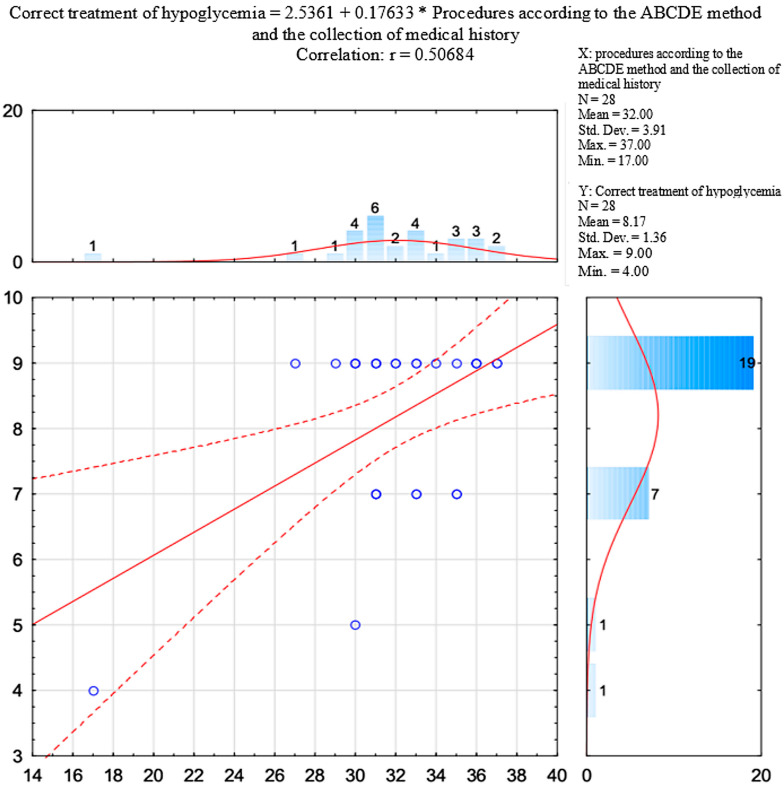
Correlation between the points obtained for examination according to the ABCDE method and the collection of medical history, and the results of assessment of the correct treatment of hypoglycaemia.

Table 5 and Figure 5 present the correlation between the points obtained for examination according to the ABCDE method and the collection of medical history, and the results of assessment of the correct treatment of hypoglycaemia.

Using the Pearson correlation coefficient, it was verified whether there was a relationship between the results of assessment of examination according to the ABCDE method and the collection of medical history and the results of assessment of the correct treatment of hypoglycaemia. The research showed a high (r = 0.507) and significant (*p* = 0.006) correlation between the analysed scales. The relationship is positive, which means that with an increase in the number of points obtained for examination according to the ABCDE method and the collection of medical history, there was greater effectiveness in the correct treatment of hypoglycaemia.

**Figure 6:**
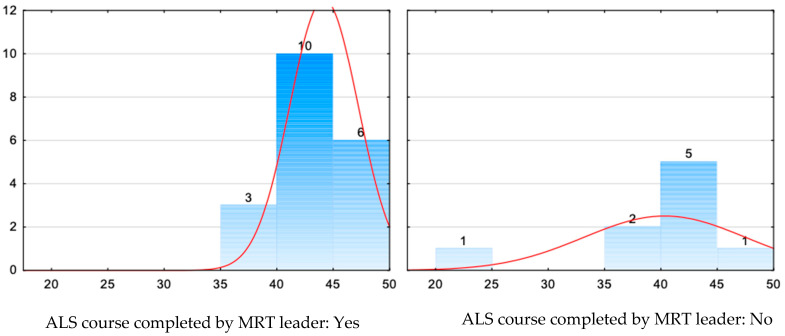
Relationship between the MRT leader having completed an ALS course and the number of points obtained.

Table 6 and Figure 6 present the relationship between the MRT leader having completed an ALS course and the number of points obtained.

The research did not show a significant relationship between the completion of an ALS course by the MRT leader and the general number of points obtained (*p* > 0.05).

**Figure 7:**
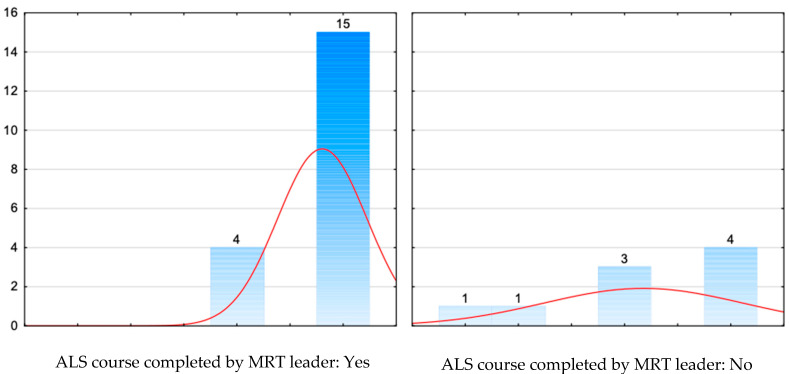
Relationship between the MRT leader having completed an ALS course and the correct treatment of hypoglycaemia.

Table 7 and Figure 7 present the relationship between the MRT leader having completed an ALS course and the correct treatment of hypoglycaemia.

When treatment performance was analysed categorically, correct management of hypoglycaemia was achieved in 79% of teams led by a provider who had completed an ALS course (15/19), compared with 44% of teams whose leader had not received ALS training (4/9). This pattern corresponded with the distribution shown in Figure 7, where ALS-trained leaders clustered at higher score values. These proportions are consistent with the result of the Mann–Whitney U test, which demonstrated a statistically significant difference between groups (U = 309.0 vs. 97.0; Z = 1.98; *p* = 0.047), indicating higher treatment scores in ALS-led teams within this specific scenario.

## 4. Discussion

One of the key elements of interventions by MRT members is the correct assessment of the location of the incident. It is at this stage that dangers to the medical personnel, patients, and witnesses can be identified, and correct assessment allows for further action to be taken [6]. Numerous studies have shown that using the correct personal protection equipment and correct assessment of the location of the incident are crucial in ensuring the safety of medical personnel [6,7]. In our research, the average number of points obtained by the teams for assessing the location of the incident was 1.86 (±0.36). As many as 86% of the MRTs obtained the maximum number of 2 points, which confirms that most teams are able to quickly and correctly assess potential dangers. Only 14% of the teams obtained a lower score (1 point), which may indicate certain shortcomings. Such deficiencies can expose MRT members to aggression [8] or infection risks [9].

Although the association between prior certified training and improved performance may appear intuitive, the present study offers several relevant contributions to the literature. First, it identifies specific domains of performance—structured assessment (ABCDE), focused history-taking (SAMPLE), teamwork, and treatment accuracy—that were most strongly associated with prior training, providing greater granularity than the general “trained versus untrained” distinction commonly reported in previous studies. Second, the use of a tightly standardised scenario allowed the identification of reproducible behavioural patterns that may support refinement of EMS training curricula. Third, the study demonstrates meaningful variability among experienced practitioners, highlighting persistent gaps in core clinical competencies that may not be easily detected through traditional assessment methods. These findings suggest that simulation-based assessment can reveal educational needs that would otherwise remain unrecognised.

Treating the patient should begin with an initial evaluation, which determines the patient’s general condition and priorities for further action. The literature shows that the high quality and correctness of this evaluation have a direct impact on correct further clinical decisions and the patient’s prognosis [10,11]. In our research, the average number of points for the initial evaluation was 5.54 (±0.69) and the maximum number of 6 points was obtained by 61% of the teams. This result reflects the teams’ high level of preparation in this area. Similar results were reported in a study analysing performance across eight editions of emergency care championships [5]. In our research, it was also shown that one team obtained only half the points for the initial evaluation. As scientific research shows, this type of error can have a negative impact on the patient’s condition [12,13]. This situation can be linked in particular to the patient’s breathing and pulse not being assessed, which can result in failure to initiate or delay in initiating mechanical ventilation or cardiopulmonary resuscitation [10,11,14].

Examining the patient is one of the most important elements of an MRT’s work, and includes assessment of their physical condition, life signs, and the identification of potential injuries. This information about the patient’s condition is an indicator for further treatment decisions implemented by the MRT [15,16]. In our research, the average number of points obtained for patient examination was 21.04 (±2.95), which shows the teams’ high, though varied, level of knowledge and skills in this area. Only one team obtained the maximum number of 26 points, while most teams (21%) obtained 20 points. The lowest score a team obtained for examining the patient was 12 points. Numerous studies confirm that insufficient or incomplete physical examination increases the risk of misdiagnosis and delays in care [15,16,17,18,19,20]. Collecting medical history according to the SAMPLE method is recommended by the ERC, as it is the basis for making the correct diagnosis [14]. In our research, the average number of points for collecting the medical history was 5.43 (±0.88). Over half the teams (57%) obtained the maximum number of 6 points, which confirms their appropriate knowledge and skills of communicating with the patients and incident witnesses. The remaining teams collected incomplete histories. The resulting lack of knowledge about the patient can result in both failure to initiate emergency care or in treatment that may not be advisable for the patient [20,21]. The information obtained from the patient about allergies or medication is crucial from the perspective of using the appropriate pharmacotherapy for them [22]. Meanwhile, information from the patient about current symptoms, past illnesses, previous operations, and the circumstances of the incident can provide suggestions for further therapeutic decisions, as well as the place the patient is transported to [23].

The last element analysed during the research was making a correct diagnosis and implementing appropriate treatment. This stage required the teams not only to have the appropriate clinical knowledge but also the skills to quickly apply suitable emergency medical care. In our research, the average number of points in this area was 8.96 (±1.53). Almost half of the teams (46%) obtained the maximum number of 10 points, which indicates a high level of competence in this area. As scientific research has shown, the poorer results of the remaining teams could be linked both to a lack of appropriate substantive preparation as well as the effect of the stress accompanying the teams during the intervention [24,25,26]. For this reason, it is vital that MRT members attend regular training that will help to improve their level of knowledge and skills regarding interventions on patients with various illnesses [27,28].

ERC guidelines emphasise that structured assessment (ABCDE, SAMPLE) is crucial for clinical decision-making, especially in emergencies [14]. In our research, none of the teams obtained the maximum of 38 points for conducting the evaluation using the ABCDE method and collecting medical history. With the exception of one, all the teams obtained between 27 and 37 points. These results show that the teams demonstrated a reasonably good level of knowledge and skills in this regard while completing the tasks. Deficiencies in this domain may negatively affect patient outcomes by delaying appropriate treatment or leading to unnecessary procedures [29,30,31].

Hypoglycaemia is a condition characterised by a low concentration of glucose in the blood. It is most frequently diagnosed in patients with diabetes, and to a large degree is caused by insufficient consumption of food or excessive intake of hypoglycemic medication [32,33]. Pre-hospital management is based on intravenous glucose administration [14]. In our research, as many as 68% of the medical response teams obtained the maximum number of 9 points for the treatment of hypoglycaemia, with the average number of points being 8.18 (±1.36). These results show that the teams taking part in the championships have a high level of skills in treating hypoglycaemia. Scientific research has shown that this type of effectively implemented pre-hospital intervention plays a key role in ensuring patients’ safety [34]. It allows serious complications to be avoided, such as convulsions [35], heart rhythm disturbances [36], and even the death of the patient [37].

Structured assessment supports accurate diagnosis and correct emergency care implementation [15]. Olgers et al. showed that the use of structured diagnostic algorithms, such as ABCDE, increases the effectiveness of interventions in emergencies as it reduces the risk of omitting key aspects of the clinical assessment [38]. In our research, this relationship was confirmed. Analysis of the teams’ interventions showed that with an increase in the number of points obtained for examining the patient and collecting medical history, there was an increase in the number of points obtained for treating hypoglycaemia. This positive relationship may be due to the fact that the ABCDE examination method and collecting medical history using SAMPLE contain elements that are useful in diagnosing hypoglycaemia. These include: determining the level of glucose in the blood, skin moisture, the level of consciousness, and the collection of information on chronic illnesses and medication taken [14].

Specialist training, including Advanced Life Support (ALS) courses, is an important element of improving the competencies of medical personnel working in the emergency medical services. Numerous studies have shown that participation in this type of course considerably improves the quality and effectiveness of interventions in emergencies, which translates into better clinical outcomes for patients [1,2,27]. In our study, teams led by a medical response team leader who had completed a certified ALS course more frequently achieved correct treatment of hypoglycaemia, with a non-significant trend towards higher overall scenario scores. These observations should be interpreted cautiously in view of the small sample size. Similar relationships have been demonstrated in other research, where the competences of the leader, especially those gained on resuscitation courses, were a key factor in the taking of correct clinical decisions in pre-hospital conditions [39,40,41]. The role of the leader is particularly important in dynamic and complex situations where rapid assessment of the patient’s condition and correct treatment decisions can be crucial for their survival. ALS courses, as well as related certified training, such as Advanced Pediatric Life Support (APLS), International Trauma Life Support (ITLS) and Prehospital Trauma Life Support (PHTLS), shape these skills by standardising knowledge and practices and developing teamwork skills [42,43]. However, training is not the only determinant of intervention quality; factors such as experience, organisational context, and individual characteristics also play important roles. Therefore, results should be interpreted with these variables in mind.

The authors are aware of certain limitations to this research. Above all, the small sample size must be mentioned, which limits the possibility of generalising the results to the wider population. Given this limited number of participating teams, the findings should be interpreted as exploratory and hypothesis-generating rather than as evidence of training efficacy. Another limitation is the heterogeneity of the study group. The participants differed in terms of professional experience, place of employment, and level of education. Although these factors were not taken into consideration in the analysis, they can have a significant impact on the level of knowledge and skills, irrespective of participation in ALS training. It is also worth underlining that the championship participants could have been selected by their units as the best representatives, which could have translated into excessively high results regarding their competencies. This means that the study group could have been characterised by an above-average level of preparation, which could have affected the assessment of the effectiveness of the training itself. Finally, although simulation provides a controlled and standardised environment for assessment, it cannot fully replicate real-world pre-hospital conditions, which further limits the generalisability of these findings.

## 5. Conclusions

The teams participating in the simulated hypoglycaemia scenario generally demonstrated a high level of knowledge and skills. More complete structured patient assessment and a more comprehensive medical history were associated with higher treatment scores, indicating that these elements contributed to better performance within this specific scenario. An observed difference was also noted between teams led by ALS-trained and non-trained leaders: correct treatment of hypoglycaemia was achieved in 79% (15/19) of ALS-led teams compared with 44% (4/9) of teams without such training. This finding, although statistically significant (U = 309.0 vs. 97.0; *p* = 0.047), should be interpreted cautiously and strictly within the context of this simulation and cannot be generalised to broader EMS practice or considered evidence of training efficacy.

## Figures and Tables

**Table 1:** Characteristics of the study group.

	Number (N)	Percentage (%)
Gender:		
Female	8	9.5
Male	75	89.3
No response	1	1.2
Age:		
20–25	11	13.1
26–35	47	56
36–45	20	23.8
Over 45	6	7.1
Education:		
Vocational school/Further education	13	15.5
Bachelor’s	48	57.1
Master’s	23	27.4
Profession:		
Paramedic	76	90.5
Nurse	2	2.4
People with the professional title of both nurse and paramedic	6	7.1
Length of work experience:		
0–5 years	24	28.6
6–15 years	44	52.4
16–25 years	16	15.5
Over 25 years	3	3.6
Place of work:		
Medical response team	84	100%
Medical transport	4	4.8%
Emergency department/admissions department	18	21.4%
Hospital wards	9	10.7%
Completed ALS course:		
Yes	19	68%
No	9	32%

**Table 2:** General results—descriptive statistics.

Elements on Assessment Cards Awarded Points	Descriptive Statistics					
	N	Mean	Median	Min.	Max.	Std. Dev.
1. Assessment of the location of the incident	28	1.86	2	1	2	0.36
1.1. Safety assessment	28	0.89	1	0	1	0.31
1.2. Personal protection equipment	28	0.96	1	0	1	0.19
2. Initial evaluation	28	5.54	6	3	6	0.69
2.1. Assessment of consciousness	28	1.00	1	1	1	0.00
2.2. Assessment on AVPU/GCS scale	28	0.64	1	0	1	0.49
2.3. Removal of contents of airways	28	1.00	1	1	1	0.00
2.4. Instrumental airway management	28	0.93	1	0	1	0.26
2.5. Assessment of breathing	28	1.00	1	1	1	0.00
2.6. Assessment of pulse	28	0.96	1	0	1	0.19
3. Patient examination	28	21.04	21	12	26	2.95
3.1. Spo2 assessment	28	0.96	1	0	1	0.19
3.2. Listening to the chest	28	1.71	2	1	2	0.46
3.3. Assessment of the skin and capillary refill	28	1.96	2	0	4	1.60
3.4. Assessment of blood pressure	28	1.00	1	1	1	0.00
3.5. Performance of 12-lead ECG	28	0.89	1	0	1	0.31
3.6. Assessment of the pupils	28	0.96	1	0	1	0.19
3.7. Assessment of glycemia	28	3.00	3	3	3	0.00
3.8. Assessment of deep temperature	28	0.89	1	0	1	0.31
3.9. Patient exposure, injury diagnosis	28	1.89	2	1	2	0.31
3.10. Repeated removal of the content of the airways	28	1.93	2	0	2	0.38
3.11. Use of a new catheter for the second removal	28	0.29	0	0	2	0.71
3.12. Selection of a second place for intraosseous access due to contraindications	28	2.89	3	0	3	0.57
3.13. Obtaining i.o. access	28	2.64	3	0	3	0.68
4. Medical history following the SAMPLE method	28	5.43	6	2	6	0.88
4.1. SAMPLE	28	5.43	6	2	6	0.88
5. Diagnosis and treatment	28	8.86	9	4	10	1.48
5.1. Administration of glucose at a minimum dose of 200 mg/kg body weight	28	4.68	5	0	5	1.19
5.2. Repeated glycemia measurement	28	3.50	4	2	4	0.88
5.3. Securing fractured lower leg	28	0.68	1	0	1	0.48
Task assessment card—overall result	28	42.82	43	23	49	4.96

**Table 3:** Assessment of the teams in terms of examination of the patient using the ABCDE method and the collection of the medical history.

Assessment Card Elements	Descriptive Statistics					
	N	Mean	Median	Minimum	Maximum	Standard Deviation
Procedures in accordance with the ABCDE method, and collecting the medical history	28	32.00	32	17	37	3.92

**Table 4:** Assessment of the teams with regard to the correct treatment of hypoglycaemia.

Assessment Card Elements	Descriptive Statistics					
	N	Mean	Median	Minimum	Maximum	Standard Deviation
Correct treatment of hypoglycaemia.	28	8.18	9	4	9	1.36

**Table 5:** Correlation between the points obtained for examination according to the ABCDE method and the collection of medical history, and the results of assessment of the correct treatment of hypoglycaemia.

Examination According to the ABCDE Method and the Collection of Medical History (Variable X) and Correct Treatment of Hypoglycaemia (Variable Y)	Procedures in Accordance with the ABCDE and SAMPLE Methods	Correct Treatment of Hypoglycaemia
Mean	32.00	8.18
Standard deviation	3.92	1.36
r (X,Y)		0.507
r2		0.257
t		2.998
***p***		**0.006**

**Table 6:** Relationship between the MRT leader having completed an ALS course and the number of points obtained.

	U Mann–Whitney Test (with Continuity Correction) In Relation to the Variable: ALS Course Completed by MRT Leader					
	Rank Sum (Yes)	Rank Sum (No)	With Correction	*p*	N (Yes)	N (No)
Total points obtained	308.5000	97.50000	1.607560	0.107933	19	9

**Table 7:** Relationship between the MRT leader having completed an ALS course and the correct treatment of hypoglycaemia.

	U Mann–Whitney Test (with Continuity Correction) In Relation to the Variable: ALS Course Completed by MRT Leader					
	Rank Sum (Yes)	Rank Sum (No)	With Correction	*p*	N (Yes)	N (No)
Correct treatment of hypoglycaemia	309.0000	97.00000	1.979251	0.047789	19	9

## Data Availability

The datasets used and/or analysed during this study are available from the corresponding author on reasonable request.
